# Biomarkers for oralization during long-term proton pump inhibitor therapy predict survival in cirrhosis

**DOI:** 10.1038/s41598-019-48352-5

**Published:** 2019-08-19

**Authors:** Angela Horvath, Florian Rainer, Mina Bashir, Bettina Leber, Bianca Schmerboeck, Ingeborg Klymiuk, Andrea Groselj-Strele, Marija Durdevic, Daniel E. Freedberg, Julian A. Abrams, Peter Fickert, Philipp Stiegler, Vanessa Stadlbauer

**Affiliations:** 1 Department of Gastroenterology and Hepatology, Medical University of Graz, Graz, Austria; 2 Center for Biomarker Research in Medicine (CBmed), Graz, Austria; 3 Department of Endocrinology and Diabetology, Medical University of Graz, Graz, Austria; 4 Department of Transplantation Surgery, Medical University of Graz, Graz, Austria; 5 Center for Medical Research, Core Facility Molecular Biology, Medical University of Graz, Graz, Austria; 6 Center for Medical Research, Core Facility Computational Bioanalytics, Medical University of Graz, Graz, Austria; 7 Division of Digestive and Liver Diseases, Columbia University Medical Center, New York, USA

**Keywords:** Liver cirrhosis, Microbiota

## Abstract

Proton pump inhibitors (PPI) are an invaluable therapy option for acid related diseases; however, PPI therapy is also linked to a series of side effects in cirrhosis, such as microbiome alterations, spontaneous bacterial peritonitis and hepatic encephalopathy. Decision tools to balance benefits and risks of PPI therapy are largely missing. In this study, we tested gut-derived biomarkers to identify PPI-associated dysbiosis, its association with gut barrier function and liver-related mortality. In this observational study, faecal microbiome composition data obtained from 16S rDNA sequencing of 90 cirrhotic patients with and without long-term PPI use and additional potential biomarkers identified from the literature were evaluated for their predictive value regarding PPI-associated dysbiosis and liver-related three-year mortality. In addition, faecal calprotectin, faecal zonulin and serum lipopolysaccharides were assessed as markers for intestinal inflammation, gut permeability and bacterial translocation. *Streptococcus salivarius*, *Veillonella parvula* and the genus *Streptococcus* were significantly increased in patients with long-term PPI therapy and performed well as biomarkers for PPI-associated dysbiosis (accuracy: 74%, 72% and 74%, respectively). The abundance of *Streptococcus salivarius* was linked to intestinal inflammation and gut barrier dysfunction, whereas the abundance of *Veillonella parvula* showed associations with liver disease severity; both were independent predictors for liver-related three-year mortality. Gut-derived biomarkers of PPI-associated dysbiosis are linked to worse outcome and a potential option to evaluate the risks of adverse events during long-term PPI therapy.

## Introduction

Proton pump inhibitors (PPI) have significantly improved the treatment of gastric acid related diseases. Since PPI have a low side effect profile, they are currently widely used, frequently without evidence-based indication, also in patients with cirrhosis^1,2^. Recent studies have raised safety concerns since the use of PPI has been linked to an increased risk of infection^2,3^, especially spontaneous bacterial peritonitis^4^, and the occurrence of hepatic encephalopathy in cirrhotic patients^5,6^. Furthermore, an increased mortality risk in patients with long-term PPI use is currently a matter of debate^7–9^.

PPI use influences the gastrointestinal microbiome: changes in the taxonomic composition and loss of alpha diversity in the distal intestine, small intestinal bacterial overgrowth and an increase in bacterial load of the gastric fluid are associated with PPI use^10–13^. Due to the increased survival rate of food-borne and oral bacteria during the gastric passage, the abundance of oral bacteria in the composition of the intestinal microbiome (i.e. oralization) is increased in patients on PPI therapy^14,15^.

The clinical relevance of PPI-associated changes in the microbiome is not yet fully elucidated. Dysbiosis in general has been linked to endotoxemia and the occurrence of infections in cirrhosis, and PPI-associated dysbiosis exacerbates NSAID-induced small intestinal injury^16,17^. Infections in patients with cirrhosis during PPI therapy are more often caused by typical gut microbes than infections without PPI involvement, which suggests that PPI use can influence the gut barrier, probably due to induction of dysbiosis and associated inflammation^18,19^. Withdrawal from PPI use can ameliorate PPI-associated dysbiosis and reduce re-hospitalization in patients with cirrhosis^20^. However, for patients that suffer from acid related diseases, withdrawal from PPI therapy is not a valid option. Procedures to balance the risk of untreated acid related diseases and the risks of PPI-associated side effects are a dire clinical need. Biomarkers for increased risks of PPI-associated side effects might help to decide whether to keep a patient with cirrhosis on PPI therapy or if withdrawal is the safer option.

In the present study, we tested microbiome derived biomarkers to identify PPI-associated dysbiosis, its association with gut barrier function and liver-related mortality.

## Results

### Changes in faecal microbiome and biomarkers

**Figure 1:**
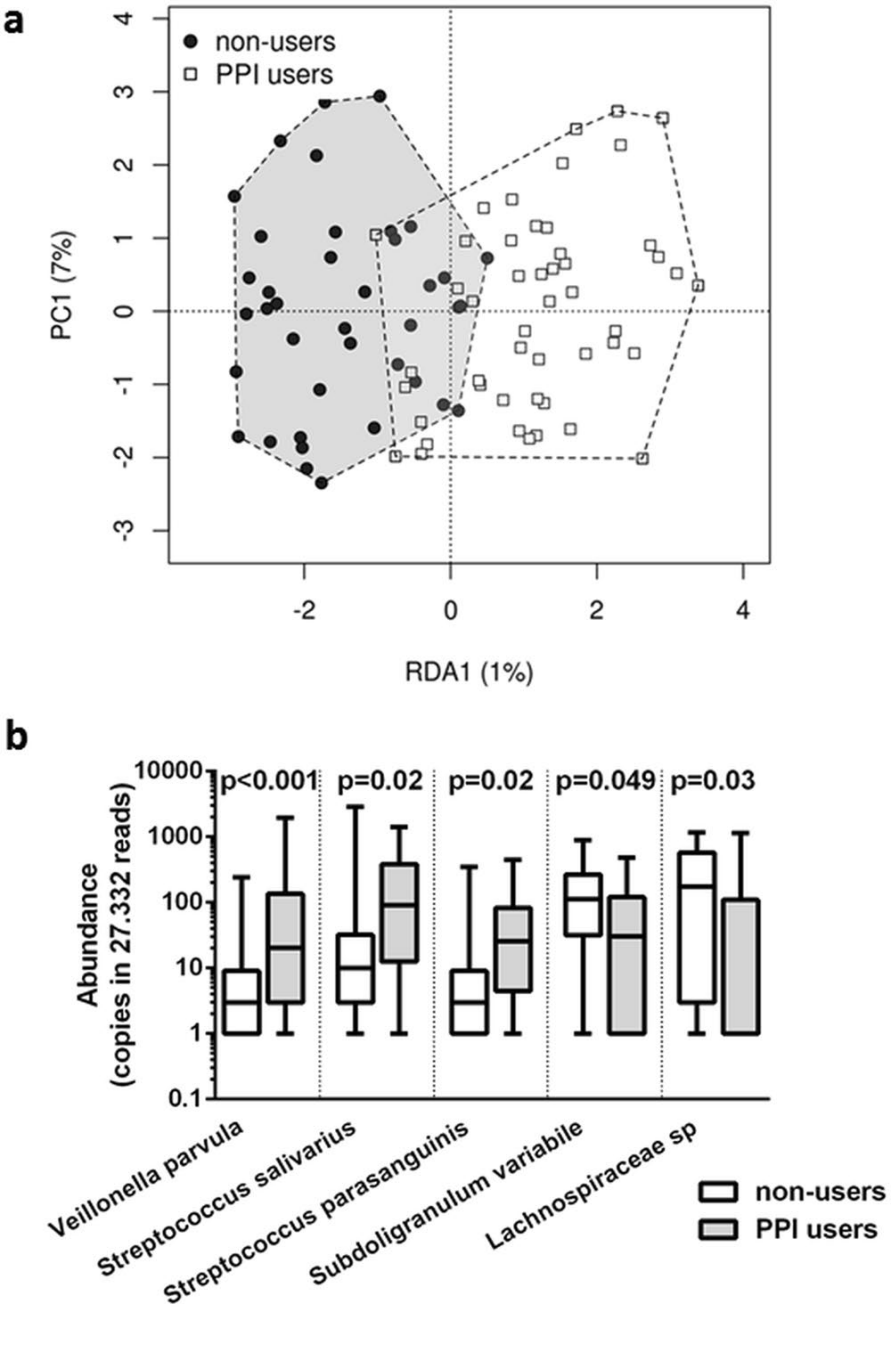
PPI-associated changes in the faecal microbiome of cirrhotic patients; (**a**) RDA-analysis based on Bray-Curtis dissimilarity matrix; (**b**) Abundance (i.e. copies in 27.332 sequencing reads) of potential biomarkers for PPI-associated dysbiosis.

Ongoing PPI use did not affect microbial diversity, but was found to significantly influence microbiome composition (p = 0.001, Fig. 1a) in patients with liver cirrhosis. In particular, patients with ongoing PPI intake showed significantly higher abundance of *Veillonella parvula, Streptococcus salivarius* and *Streptococcus parasanguinis* (all commonly found in the oral cavity), and lower abundance of *Subdoligranulum variabile* and a not further classified *Lachnospiraceae* species compared to patients without PPI intake. (For details see Fig. 1b and Supplementary Table 2)

**Figure 2:**
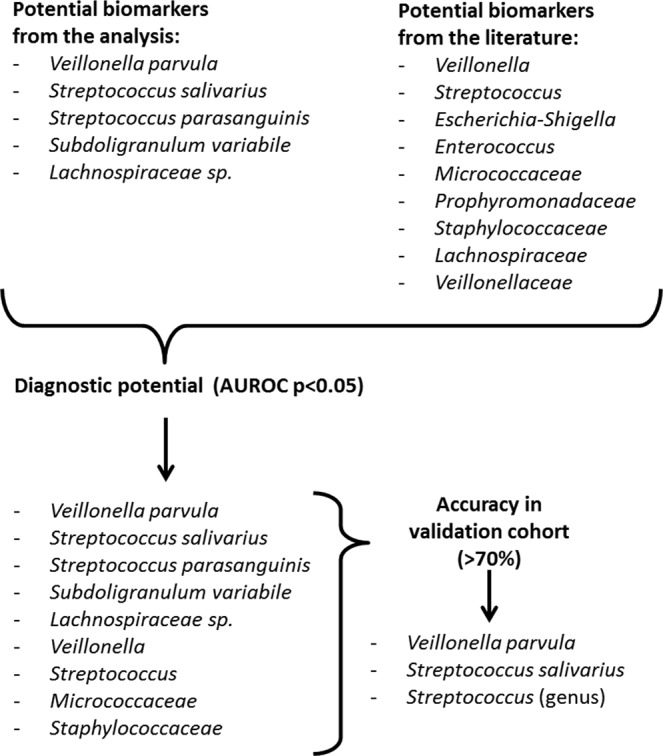
Workflow for biomarker identification.

Together with bacterial taxa that have been implicated in PPI-associated dysbiosis in previous work (i.e. *Micrococcaceae*, *Porphyromonadaceae, Staphylococcaceae*, *Lachnospiraceae*, *Veillonellaceae*, *Streptococcus*, *Escherichia-Shigella*, *Enterococcus* and *Veillonella*^13–15,20,21^), predictive merit and accuracy was assessed. Initially, fourteen taxa were screened. Of those taxa, nine could sufficiently allocate patients to PPI use or non-use (AUROC - p < 0.05: the species *Veillonella parvula*, *Streptococcus salivarius*, *Streptococcus parasanguinis*, *Lachnospiraceae sp*. and *Subdoligranulum variabile*, the genera *Streptococcus* and *Veillonella*, and the families *Micrococcaceae* and *Staphylococcaceae*). Those nine taxa were then further tested in a cirrhotic validation cohort (a subset of the original cohort with a one year interval between sample collections). *Veillonella parvula*, *Streptococcus salivarius* and the genus *Streptococcus* could predict PPI use in the validation cohort with an accuracy of at least 70%. (An overview is given in Fig. 2, for details see Supplementary Table 2) The spectrum of dysbiosis combinations is given in Supplementary Table 3.

### Mortality risk

Patients with and without PPI use were matched according to Child-Pugh and MELD scores; 50 pairs were analysed with a median coefficient of variation in propensity scores of 0.43% (0.31; 0.68). Accordingly, liver disease severity was well comparable between groups (Table 1). Outcome data was collected for a median time of 36 months. Ten percent of patients (10/100) died within this period, nine of which were on long-term PPI therapy.

**Table 1:** Patients characteristics for propensity score matched pairs (n = 50). Data is given as median and 95% confidence interval.

	PPI	No PPI	p-value
Age (years)	58 (56–62)	55 (53–60)	0.117
Sex (female/male)	10/40 (20%/80%)	13/37 (26%/74%)	0.635
Child-Pugh score	6 (5–6)	6 (5–7)	0.895
MELD score	11 (10–13)	11.5 (10–13)	0.669
Antibiotic use	2 (4%)	0 (0%)	0.495
Metformin	8 (16%)	5 (10%)	0.554
Lactulose	8 (16%)	2 (4%)	0.091

PPI: proton pump inhibitor.

**Figure 3:**
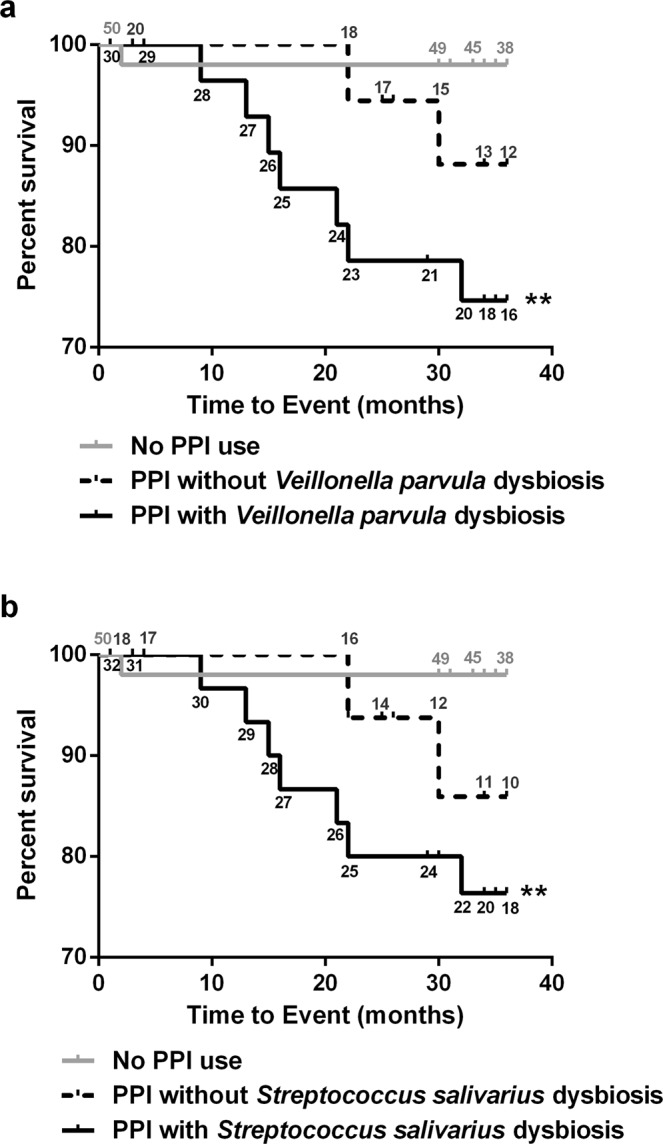
Biomarkers for PPI-associated dysbiosis predict liver-related three-year mortality. Kaplan-Meier curves for three-year survival according to PPI use and dysbiosis defined by (**a**) *Veillonella parvula* abundance and (**b**) *Streptococcus salivarius*. Patients at risk are given along the curves; patients at risk without PPI use are given in grey numbers; patients at risk with PPI without dysbiosis are given in black above the curve; patients at risk with PPI and dysbiosis are given in black below the curve; **p < 0.01 compared to patients without PPI.

Patients with PPI intake and *Veillonella parvula* dysbiosis showed significantly higher liver-related mortality compared to patients without PPI use [HR: 14.0 (95% CI: 1.7–114.1), p = 0.014], while PPI users without dysbiosis did not [HR: 5.7 (95% CI: 0.5–62.9), p = 0.155]. Same is true for patients with PPI use and *Streptococcus salivarius* dysbiosis [HR: 12.9 (95% CI: 1.6–105.1), p = 0.017 and 6.5 (95% CI: 0.6–71.4), p = 0.128, respectively]. Patients with and without *Streptococcus* (genus) dysbiosis showed significantly higher liver-related mortality compared to patients without PPI use [HR: 9.6 (95% CI: 1.1–82.5), p = 0.039 and 12.1 (95% CI: 1.3–108.1), p = 0.026, respectively]. (Fig. 3).

Also, Child-Pugh score and MELD score were predictors of liver-related mortality in patients with liver cirrhosis [HR: 2.0 (95% CI: 1.3–2.9), p = 0.001 and 1.2 (95% CI: 1.0–1.4), p = 0.015, respectively]. Therefore, multivariate Cox-Regression was used to account for their confounding influence. *Veillonella parvula* dysbiosis and *Streptococcus salivarius* dysbiosis, but not *Streptococcus* (genus) dysbiosis were found to be independent predictors for liver-related mortality (for details see Table 2).

**Table 2:** Hazard ratios (liver-related three-year mortality) for PPI-associated dysbiosis adjusted for liver disease by multivariate Cox-Regression.

Biomarkers	Hazard ratio	95% CI	p-value
**Model 1: Liver function and** ***Veillonella parvula*** **dysbiosis**			
Child-Pugh score	3.2	1.2–8.3	**0.018**
MELD score	0.8	0.5–1.1	0.167
PPI use with Veillonella parvula dysbiosis	14.6	1.7–122.2	**0.013**
PPI use without Veillonella parvula dysbiosis	4.1	0.4–47.2	0.263
**Model 2: Liver function and** ***Streptococcus salivarius*** **dysbiosis**			
Child-Pugh score	2.4	1.1–5.6	**0.035**
MELD score	0.9	0.6–1.2	0.350
PPI use with Streptococcus salivarius dysbiosis	10.7	1.3–89.3	**0.029**
PPI use without Streptococcus salivarius dysbiosis	6.8	0.6–77.2	0.120
**Model 3: Liver function and** ***Streptococcus*** **(genus) dysbiosis**			
Child-Pugh score	3.0	1.2–7.8	**0.022**
MELD score	0.8	0.6–1.1	0.213
PPI use with Streptococcus (genus) dysbiosis	6.5	0.7–58.2	0.092
PPI use without Streptococcus (genus) dysbiosis	18.6	2.0–174.5	**0.011**

CI: confidence interval; MELD: model of end stage liver disease; PPI: proton pump inhibitor.

### Gut barrier function

Patients with *Streptococcus salivarius* dysbiosis showed significantly higher median faecal calprotectin levels than PPI users without dysbiosis as well as patients without PPI use (p = 0.012 and p < 0.001, respectively). Furthermore, those patients showed significantly higher faecal zonulin and serum lipopolysaccharide (LPS) levels compared to patients without PPI use (p = 0.019 and p = 0.032, respectively).

Patients with PPI use, irrespective of *Veillonella parvula* or *Streptococcus* (genus) dysbiosis, showed significantly higher faecal calprotectin levels compared to patients without PPI use (p < 0.001 and p < 0.001, as well as p = 0.005 and p < 0.001, respectively). Patients with *Veillonella parvula* dysbiosis showed higher faecal zonulin and serum lipopolysaccharide (LPS) levels compared to patients without PPI use (p = 0.035 and p = 0.009, respectively). LPS levels were also higher in patients without *Streptococcus* (genus) dysbiosis compared to patients without PPI use (p = 0.025). Details are given in Table 3.

**Table 3:** Faecal calprotectin, faecal zonulin and serum LPS for patients with PPI-associated dysbiosis. Data is given as Median and 95% confidence interval.

	PPI use with dysbiosis	PPI use without dysbiosis	No PPI use	Adjusted p-value
***Streptococcus salivarius*** **dysbiosis**				
N	32	18	50	
Calprotectin (ng/mg)	271 (206–394)	59 (32–206)	17 (12–65)	^**a**^**p** < **0.001**, ^b^p = 0.059, ^**c**^**p** = **0.012**
Zonulin (ng/mg)	85 (71–102)	83 (62–97)	69 (51–78)	^**a**^**p** = **0.019**, ^b^p = 0.165, ^c^p > 0.999
LPS (EU/ml)	3 (0–9)	0 (2–7)	0 (0–1)	^**a**^**p** = **0.032**, ^b^p = 0.116, ^c^p > 0.999
***Veillonella parvula*** **dysbiosis**				
N	30	20	50	
Calprotectin (ng/mg)	218 (167–348)	206 (48–318)	17 (12–65)	^**a**^**p** < **0.001**, ^**b**^**p** < **0.001**, ^c^p = 0.961
Zonulin (ng/mg)	84 (65–100)	84 (71–102)	69 (51–78)	^**a**^**p** = **0.035**, ^b^p = 0.074, ^c^p > 0.999
LPS (EU/ml)	3 (1–12)	1 (0–7)	0 (0–1)	^**a**^**p = 0.009**, ^b^p = 0.333, ^c^p > 0.999
***Streptococcus*** **(genus) dysbiosis**				
N	31	19	50	
Calprotectin (ng/mg)	270 (183–406)	167 (32–264)	17 (12–65)	^**a**^**p** = **0.005**, ^**b**^**p** < **0.001**, ^c^p = 0.100
Zonulin (ng/mg)	90 (71–109)	83 (47–97)	69 (51–78)	^**a**^**p** = **0.005**, ^b^p = 0.465, ^c^p = 0.710
LPS (EU/ml)	2 (0–7)	3 (0–12)	0 (0–1)	^a^p = 0.096, ^**b**^**p** = **0.025**, ^c^p > 0.999

PPI: proton pump inhibitor; ^a^denotes differences between patients with PPI use with dysbiosis and patients without PPI use; ^b^denotes differences between patients with PPI use without dysbiosis and patients without PPI use; ^c^denotes differences between patients with PPI use with and without dysbiosis.

### Associations of *Veillonella parvula* with liver disease

*Veillonella parvula* abundance was positively correlated with MELD-score (r_s_ = 0.319, p = 0.024) and Child-Pugh score (r_s_ = 0.395, p = 0.005) in patients with PPI intake. Although there were no differences in liver function between patients with and without PPI use, associations between *Veillonella parvula* abundance and liver function were absent in patients without PPI use. *Streptococcus* (genus) and *Streptococcus salivarius* did not show significant associations to liver function. Accordingly, *Veillonella parvula* showed low accuracy in predicting PPI use in patients with end stage renal disease (ESRD) and healthy controls (67% and 54%, respectively), while *Streptococcus* (genus) and *Streptococcus salivarius* showed markedly higher accuracy in those cohorts (83% and 83%, as well as 70% and 83%, respectively).

## Discussion

Biomarkers for PPI-associated dysbiosis can be used to predict increased risk for liver-related mortality and are associated with gut barrier dysfunction in liver cirrhosis patients. *Streptococcus salivarius* abundance was especially linked to intestinal inflammation and gut permeability and *Veillonella parvula* abundance to liver function. Both were able to independently predict liver-related mortality.

*Streptococcus salivarius* and *Veillonella parvula* are bacteria typically found in the oral cavity. *Streptococcus salivarius* is a dominant species on the dorsum of the tongue and the pharyngeal mucosa^22^. Although its immunomodulating properties are often described as anti-inflammatory, it can induce pro-inflammatory cytokine secretion in oral mucosal cells, including IL-6, CXCL8 and TNFα^23–25^. *Veillonella parvula* is the most prevalent *Veillonella* species in subgingival plaques, and forms biofilms with various *Streptococcus* species including *Streptococcus salivarius*^26,27^. *Veillonella parvula* produces a lipopolysaccharide that potently induces pro-inflammatory cytokine release in peripheral blood mononuclear cells and can also induce pro-inflammatory reactions in dendritic cells, including TNFα and CXCL8 production^28,29^. CXCL8 is a chemoattractant for neutrophil granulocytes which in turn are calprotectin producers^30,31^. This is in accordance to the highly elevated calprotectin levels found in patients with PPI-associated dysbiosis in our study. TNFα-mediated inflammation has been shown to disrupt tight junctions and increase gut permeability and bacterial translocation^32^. This is reflected in the increased zonulin and LPS levels in patients with PPI-associated dysbiosis. As biomarkers, they show higher specificity than sensitivity. This suggests that PPI use is associated with a specific type of dysbiosis but not all patients with PPI use are susceptible to these changes and associated changes in gut permeability and liver-related mortality risk.

Implications of PPI use on intestinal microbiota, including increased colonization by bacteria typically found in the oral cavity, have been described recently^10,14,15,21^, and are likely the result of decreased gastric clearance of microbes due to inhibition of acid secretion^2,13^. Qin *et al*. have suggested an increase in *Veillonella parvula* abundance to be present in patients with liver cirrhosis in general^33^; however, the influence of PPI intake was not taken into account in this paper^34^. Our findings show that *Veillonella parvula* abundance is not only dependent on PPI intake but also correlates with liver disease severity. The microbiome of cirrhotic patients might also be influenced by other drugs including antibiotics, antidiabetics or laxatives and synergistic effects have to be considered. Appropriately powered studies are needed to address this issue.

In a recent study by Bajaj *et al*. it was shown that the disruption of the microbiome by PPI was partly reversible when PPI use was stopped and that withdrawal from PPI therapy prolonged time to readmission^20^. Early biomarkers, as presented in this study, might help to determine when withdrawal is necessary. Monitoring *Veillonella parvula* and *Streptococcus salivarius* in patients with liver cirrhosis during long-term PPI therapy could help to manage the risk of serious PPI side effects in these patients. It remains to be shown whether the regression of the dysbiosis also ameliorates gut barrier dysfunction and other PPI-associated side effects. If so, targeted strategies to reduce PPI-associated dysbiosis, could potentially reduce the risk of PPI side effects in cirrhotic patients when PPI therapy needs to be continued.

Biomarkers should be easily accessible and applicable for longitudinal observations. We used 16S sequencing which became widely accessible in the last years and increasingly affordable. However, this setting is not ideal for fast and accessible screening. Therefore, other PCR-based methods could be used to track the abundance of the described bacteria. These biomarkers make it possible to observe one or two operational taxonomic units (OTUs) instead of relying on the entire microbiome composition and the necessary bioinformatic analysis. Easy access also relates to the samples in which the biomarkers are measured. Faecal matter is abundant and samples can be taken without invasive procedures. However, it has to be stated that stool might vary considerable from the lumen or mucosa in its microbial composition, and that our results and biomarkers are therefore restricted to the faecal microbiome.

A noteworthy limitation of our study is the observational design. Since patients were not randomized to PPI use but rather reflected real-life prescription habits in Austria, patients with more severe liver disease (according to MELD score) were more likely to take PPI. With increasing evidence of the harmful effects of PPI intake, a randomized controlled trial would not be ethical. Therefore propensity score matching with replacements was applied, to account for differences in liver function and liver disease was acknowledged as confounder in survival analysis. The patients were also rather heterogeneous in disease aetiology, duration, preparation and dosage of PPI therapy, as well as other concomitant medication. Therefore, calculated risks are variable and other confounding variables need to be addressed in more specified studies. The variation of the microbiome in different geographical locations might limit the generalization of our biomarkers which were established in an Austrian/European cohort and tested on a US-American group of volunteers before and after a four-week PPI regime. Further studies are warranted to validate and/or refine those biomarkers on a global scale.

In conclusion, gut-derived biomarkers could become a valuable tool to evaluate the safety of PPI use in patients with cirrhosis. High *Veillonella parvula* and *Streptococcus salivarius* abundances are associated with worse outcome and therefore, might encourage a more stringent re-evaluation of PPI therapy in cirrhotic patients.

## Methods

### Patients

A dataset of 50 patients with cirrhosis on long-term PPI therapy and 40 control patients with cirrhosis without PPI therapy were the basis for this study cohort. Nearest neighbour propensity score matching with replacements was performed (i.e. one control could be used for more than one case^35^) to match each patient with PPI intake to a patient without PPI intake as a control according to liver disease severity, creating 50 patient pairs. Non-matched controls were excluded from analysis. Data was taken from baseline examinations of an intervention study that was performed between July 2012 and September 2014 at the Medical University of Graz^36^. All patients were 18 years or older, had a Child-Pugh score of 11 or lower and had given written informed consent. Patients with active alcohol abuse, active infections or gastrointestinal bleeding two weeks prior to inclusion, as well as patients with immuno-modulating drugs, hepatic encephalopathy stage two or higher, renal failure (creatinine over 1.7 mg/dL), severe diseases unrelated to cirrhosis, malignancy or pregnancy were excluded. A subset of these patients (n = 68) were sampled again after one year and used as validation cohort for biomarker identification. Ten percent of patients either started or stopped PPI therapy between samplings. The study was approved by the institutional ethics committee of the Medical University of Graz (23–096 ex 10/11), registered at clinicaltrials.gov (NCT01607528, 30.5.2012) and performed in accordance with the Declaration of Helsinki. The microbiome sequencing data was made publicly available on NCBI sequencing Read Archive (SRP132827).

To test whether identified biomarkers are restricted to liver cirrhosis, published sequencing data from liver-healthy cohorts was used. Firstly, patients with end-stage renal disease (ESRD) with (n = 14) or without (n = 16) long term PPI use were taken from NCT01362569 (30.5.2011). The sequencing data are available from the NCBI Sequence Read Archive (accession number PRJNA390475)^37^. Secondly, healthy volunteers (n = 12) before and after a four week PPI therapy from the study NCT01901276 (17.07.2013) were re-analysed^10^. Patients characteristics are given in Supplementary Table 1.

### PPI use

Long-term PPI use was defined as regular intake of PPI longer than 4 weeks, PPI non-use as absence of PPI intake for at least 4 weeks prior to study inclusion. Indications for PPI use were documented as follows: (1) gastro-oesophageal reflux disease (GERD) including Barrett’s oesophagus, (2) evidence of inflammation (esophagitis, gastritis or duodenitis) on previous endoscopies, (3) gastric or duodenal ulcers on previous endoscopies or (4) medication requiring PPI prophylaxis (like NSAIDs and steroids).

### Follow-up

Liver related mortality was documented from patients’ physicians’ or hospital records. Observation period was three years and patients were censored at liver transplantation or lost to follow-up.

### Microbiome analysis

Stool samples were collected by the patients in sterile collection tubes either on the same day or the evening before the study visit. Samples were kept on 4 °C until arrival at the hospital and then frozen immediately at −80 °C. DNA was isolated with the MagNA Pure LC DNA Isolation Kit III (Bacteria, Fungi) (Roche, Mannheim, Germany) according to manufacturer’s instructions. Hypervariable region V1–2 was amplified (primers: 27F-AGAGTTTGATCCTGGCTCAG; R357-CTGCTGCCTYCCGTA) and sequenced using Illumina Miseq technology (Illumina, Eindhoven, Netherlands) as described before^37^. Paired end reads were then joined by fastq-join tool. Primers were removed by cutadapt 1.6 and USEARCH 6.1^38^ was used for reference based chimera detection. Open reference operational taxonomic unit (OTU) picking was done with SILVA v123 as reference database. When necessary, sequences were blasted in the NCBI database for further classification^39^. Clustering was performed by UCLUST^40^ with a 97% sequence similarity threshold. Fasttree was used to generate a phylogenetic tree. After pre-processing, an average of 85.960 (range from 27.332 to 290.494) reads per sample was available. Data was normalized by rarefication (sampling depth: 27.332 reads). Data analysis was performed with QIIME1.9.1^41^ implemented on a local Galaxy instance (https://galaxy.medunigraz.at/), redundancy analysis (RDA) was done in the web-based software Calypso 8.84 (http://cgenome.net/wiki/index.php/Calypso)^42^.

### Biomarker identification and validation

For biomarker identification, low abundant operational taxonomic units (OTUs; i.e. bacterial taxa with similar 16S sequences) that were only present in one patient or did not reach 0.05% of overall abundance were excluded from analysis. Data was rarefied (sequencing depth: 16.703 reads) and the abundance of the remaining OTUs were compared between patients with and without PPI use by multiple Mann-Whitney tests with Benjamini-Hochberg correction. Supervised machine learning (Random Forest Algorithm) was used to rank OTUs according to their diagnostic potential (i.e. mean decrease on accuracy). OTUs that ranked in the top 5% and showed a statistical significant difference between patients with and without PPI intake were considered to be suitable biomarkers. Additionally, bacterial taxa already reported in the literature to be altered during PPI therapy in either the general population or in cirrhotic patients were evaluated: *Micrococcaceae*, *Porphyromonadaceae, Staphylococcaceae*, *Lachnospiraceae*, *Veillonellaceae*, *Streptococcus*, *Escherichia-Shigella*, *Enterococcus* and *Veillonella*^13–15,20,21^.

Areas under the receiver operated characteristics curve (AUROC) were calculated for all the selected biomarkers for PPI use. Based on AUROC coordinates, Youden index^43^ was calculated for various thresholds and the best performing thresholds (highest sum of specificity and sensitivity) for every biomarker were then tested in the cirrhosis validation cohort. Biomarkers that reached at least 70% accuracy in the cirrhosis validation cohort were then considered for further analysis. According to these biomarkers, patients were categorized into three groups: (1) no PPI use, (2) PPI use without dysbiosis (i.e. false negatives) and (3) PPI use with dysbiosis (i.e. true positives).

### Laboratory measurements

Faecal calprotectin, faecal zonulin and lipopolysaccharide in serum (LPS) were measured as markers for intestinal inflammation, gut permeability and bacterial translocation, respectively. Calprotectin and zonulin were assessed with a commercially available ELISA kit according to manufacturer’s instructions (Immundiognostik, Bensheim, Germany) and LPS was measured with a cell-based detection system (Invitrogen, Toulouse, France) with an adapted protocol^36^.

### Statistical analysis

Count data is presented as absolute numbers and percentages, continuous data as medians and 95% confidence intervals. Differences between groups in categorical data were evaluated with Chi-square tests and differences between groups in continuous data were evaluated with Kruskal-Wallis tests and Mann-Whitney tests with Bonferroni correction as post hoc tests. Associations between two parameters were evaluated with Spearman correlation.

Liver-related three-year mortality was analysed with Kaplan-Meier curves and Cox-Regression. To determine whether predictions based on microbiome-derived biomarkers are independent of liver function, multivariate Cox-regression with forced entry was used. MELD-score and Child-Pugh-score were used as potential confounders. P-values lower than 0.05 were considered significant.

SPSS 23 (SPSS Inc., Chicago, IL, USA) was used for statistical analysis and GraphPad Prism 6 (GraphPad Software, San Diego, USA) for visualization.

## Supplementary information

Supplementary Information

## Data Availability

The microbiome sequencing data was made publicly available on NCBI sequencing Read Archive (SRP132827). The remaining datasets analysed during the current study are available from the corresponding author on reasonable request.
