# Supplementary material for: Biomarkers for oralization during long-term proton pump inhibitor therapy predict survival in cirrhosis
Source: Sci Rep. 2019 Aug 19;9:12000. doi: 10.1038/s41598-019-48352-5 (PMC6700098; doi:10.1038/s41598-019-48352-5)
Supplement: Supplementary file 1 — Supplementary Information [file 41598_2019_48352_MOESM1_ESM.pdf]

Biomarkers for oralization during long-term proton pump inhibitor therapy predict survival in cirrhosis

Supplementary information

Angela Horvath,[1,2†*] Florian Rainer,[1†] Mina Bashir,[3] Bettina Leber,[4] Bianca Schmerboeck,[1,2] Ingeborg Klymiuk,[5] Andrea Groselj-Strele,[6] Marija Durdevic,[6] Daniel E. Freedberg,[7] Julian A. Abrams,[7] Peter Fickert,[1] Philipp Stiegler,[4] Vanessa Stadlbauer[1]

†contributed equally

[1]Department of Gastroenterology and Hepatology, Medical University of Graz, Graz, Austria

[2]Center for Biomarker Research in Medicine (CBmed), Graz, Austria

[3]Department of Endocrinology and Diabetology, Medical University of Graz, Graz, Austria

[4]Department of Transplantation Surgery, Medical University of Graz, Graz, Austria

[5]Center for Medical Research, Core Facility Molecular Biology, Medical University of Graz, Graz, Austria

[6]Center for Medical Research, Core Facility Computational Bioanalytics, Medical University of Graz, Graz, Austria

[7]Division of Digestive and Liver Diseases, Columbia University Medical Center, New York, USA

*Correspondence to angela.horvath@medunigraz.at

Methods

Non-cirrhotic validation cohorts

Two validation cohorts were established from published data to validate the bacterial taxa identified as biomarkers for PPI-associated dysbiosis in cirrhosis.[1, 2] DNA was extracted from a single stool sample and 16S rRNA gene-derived sequencing reads were analyzed as described above.

The fist validation cohort comprised 30 patients with end-stage renal disease (ESRD) undergoing haemodialysis or peritoneal dialysis with or without concomitant long-term PPI use. Patients were recruited at the University Hospital in Graz, were 18 years or older, and did not have malignancy, pregnancy, chronic inflammatory bowel disease, celiac disease, active alcohol abuse, severe organ dysfunction unrelated to renal dysfunction, or clinical evidence of active infection.[1]

The second validation cohort comprised 12 healthy volunteers before and after a four week omeprazole regime with a twice daily dose of 40mg. The participants were recruited at the Columbia University Medical center, were 18 years or older and did not match any of the exclusion criteria: use of systemic antibiotics within one year, use of PPIs or histamine-2 receptor antagonists within one year, new medications within one month, chronic gastrointestinal mucosal disease, abnormal bowel frequency (minimum of three bowel movements per week, maximum of three per day), use of medications with potential interactions with PPIs, pregnancy, and travel planned outside of the United States during the study period.[2]

Characteristics are given in Supplementary Table 1.

Supplementary Tables:

Supplementary Table 1. Patient characteristics for validation cohorts. Data is given as count (%) or median and 95% confidence interval.

	healthy	end stage renal disease	
		no PPI	PPI
N	12	16	14
Age (in years)	39 (28 - 52)	61 (54 - 72)	61 (53 - 68)
Gender (m/f)	6/9	10/6	12/2
Antibiotic treatment	0 (0%)	1 (6%)	0 (0%)
Antidiabetic therapy	1 (8%)	3 (19%)	0 (0%)
- Metformin	1 (8%)	0 (0%)	0 (0%)

Supplementary Table 2. Biomarkers from present analysis or literature with abundance between PPI users and non-users, as well as biomarker performance in training (n=100) and validation cohorts (n=68) of cirrhotic patients.

		Abundance (Median and 95% CI)			Training			Validation		
Biomarker	Origin	PPI	No PPI	p-value	AUROC (SE)	p-value	Threshold	Accuracy (%)	Sensitivity	Specificity
**Species**										
Veillonella parvula	*A*	**20.5 (5 - 61)**	**1.5 (0 - 4)**	**<0.001**	**0.742 (0.052)**	**<0.001**	**10**	**72**	**0.63**	**0.85**
Streptococcus salivarius	*A*	**91.5 (41 - 177)**	**10.5 (6 - 26)**	**0.02**	**0.717 (0.055)**	**<0.001**	**42**	**74**	**0.71**	**0.78**
Streptococcus parasanguinis	*A*	**25.5 (13 - 36)**	**3.5 (2 - 9)**	**0.02**	**0.709 (0.055)**	**0.001**	**14**	65	0.56	0.78
Lachnospiraceae sp.	*A*	**0 (0 - 1)**	**52.5 (2 - 203)**	**0.03**	**0.673 (0.058)**	**0.003**	**7**	60	0.37	0.41
Subdoligranulum variabile	*A*	**30.5 (3 - 86)**	**109.5 (47 - 162)**	**0.049**	**0.642 (0.059)**	**0.019**	**37**	54	0.46	0.44
**Genera**										
Streptococcus	*L*	**334 (162 - 455)**	**67.5 (27 - 126)**	**0.001**	**0.740 (0.052)**	**<0.001**	**252**	**74**	**0.63**	**0.89**
Veillonella	*L*	**53 (17 - 202)**	**3.5 (1 - 14)**	**<0.001**	**0.721 (0.055)**	**<0.001**	**34**	68	0.54	0.89
Enterococcus	*L*	0 (0 - 2)	0 (0 - 0)	0.8	0.564 (0.060)	0.236	33	-	-	-
Escherichia-Shigella	*L*	5 (1 - 10)	1.5 (0 - 13)	0.8	0.550 (0.061)	0.400	206	-	-	-
Families										
Micrococcaceae	*L*	**6 (3 - 9)**	**1 (1 - 2)**	**0.001**	**0.746 (0.053)**	**<0.001**	**2**	68	0.71	0.63
Staphylococcaceae	*L*	0 (0 - 0)	0 (0 - 0)	0.1	**0.570 (0.060)**	**0.013**	**1**	50	0.10	0.96
Veillonellaceae	*L*	356 (214 - 531)	94.5 (28 - 582)	0.1	0.594 (0.061)	0.124	33	-	-	-
Porphyromonadaceae	*L*	578 (255 - 910)	803.5 (540 - 1192)	0.4	0.421 (0.061)	0.196	15	-	-	-
Lachnospiraceae	*L*	6700 (6097 - 7365)	6718.5 (6319 - 7547)	0.4	0.466 (0.061)	0.582	6940	-	-	-

CI: confidence interval - PPI: proton pump inhibitor - AUROC: Area under the receiver operated curve - SE: standard error - A: from analysis - L: from literature

Supplementary Table 3. Spectrum of dysbiosis combinations among cirrhotic patients with PPI use.

**Types of dysbiosis**	**N (%)**
No dysbiosis	8 (16%)
Veillonella parvula dysbiosis	6 (12%)
Streptococcus salivarius dysbiosis	1 (2%)
Streptococcus (genus) dysbiosis	4 (8%)
Veillonella parvula + *Streptococcus salivarius* dysbiosis	4 (8%)
Veillonella parvula + *Streptococcus (genus)* dysbiosis	0 (0%)
Streptococcus salivarius + *Streptococcus* (genus) dysbiosis	7 (14%)
Veillonella parvula + *Streptococcus salivarius* + *Streptococcus* (genus) dysbiosis	20 (40%)

Supplementary References

1. Stadlbauer V, Horvath A, Ribitsch W, et al. Structural and functional differences in gut microbiome composition in patients undergoing haemodialysis or peritoneal dialysis. Scientific reports. 2017;7(1):15601. Epub 2017/11/17.
2. Freedberg DE, Toussaint NC, Chen SP, et al. Proton Pump Inhibitors Alter Specific Taxa in the Human Gastrointestinal Microbiome: A Crossover Trial. Gastroenterology. 2015;149(4):883-5.e9. Epub 2015/07/15.
